# Electrochemical Deposition and Formation Mechanism of Single-Crystalline Cu_2_O Octahedra on Aluminum

**DOI:** 10.1155/2012/406162

**Published:** 2012-06-12

**Authors:** Q. T. Du, J. S. Tan, Q. T. Wang, C. Y. Li, X. H. Liu, R. S. Cai, Y. H. Ding, Y. Q. Wang

**Affiliations:** ^1^Cultivation Base for State Key Laboratory, Qingdao University, No. 308 Ningxia Road, Qingdao 266071, China; ^2^College of Physics Science, Qingdao University, No. 308 Ningxia Road, Qingdao 266071, China

## Abstract

A simple electrochemical deposition was developed to synthesize the cuprous oxide (Cu_2_O) octahedra on aluminum foils. The average edge length of the octahedra is about 300 nm. The chemical composition of the octahedra was determined using energy dispersive X-ray spectroscopy and electron energy-loss spectroscopy. The microstructure of the octahedra was investigated using transmission electron microscopy. The formation mechanism of the octahedra is proposed.

## 1. Introduction

It is well known that the shape and size of the inorganic nanoparticles have a great influence on their physical properties [1]. In recent years, the synthesis of nanoparticles with well-controlled morphology and size has received lots of interest for their potential applications in nanoscale plasmon photonics, drug delivery, calorimetric sensing [2], nanoelectronics, catalysis, and information storage [3–5]. Two general strategies are usually employed for the “bottom-up” chemical synthesis of nanomaterials: one is the use of the templates, which could confine the shape and size of nanoparticles [6]; the other is the use of the capping agents, which could control the direction and dimension of nanoparticles during the growth process [7, 8].

Cuprous oxide (Cu_2_O), which has excellent optical and magnetic properties, is a relatively nontoxic p-type semiconductor with a direct bandgap of 2.17 eV [9]. Tailoring the architecture of Cu_2_O crystals has received extensive attention. Over the past few decades, a variety of well-controlled Cu_2_O micro-/nanostructures such as cubes [10–15], octahedra [16–19], dodecahedra [17], nanowires [20], and hollow spheres [21] have been synthesized by various methods. Comparing with other methods, electrochemical deposition is a convenient way of preparing many well-defined structures with high uniformity. It has been reported that Cu_2_O could be deposited onto different substrates such as Pt, Cu, Au, indium-doped tin oxide (ITO), and stainless steel using electrochemical method [22], most of which concentrated on the fabrication of Cu_2_O films or nanowires. Three main methods have been reported to prepare Cu_2_O octahedra (a) solution-phase route with surfactants; (b) reduction method with glucose, hydrazine hydrate, and ascorbic acid; (c) *γ*-irradiation with Triton X-100 water-in-oil microemulsions. Few reports can be found to synthesize Cu_2_O octahedra by electrochemical deposition onto the Al foils with the existence of capping agents, and the formation mechanism of Cu_2_O octahedra is not clear yet. 

Here, we report the electrochemical deposition of single-crystalline Cu_2_O octahedra onto aluminum (Al) foils using alkaline cupric sulfate solutions stabilized by lactate ions. Morphology and microstructure of Cu_2_O octahedra were investigated in detail. The formation mechanism of the octahedra is proposed.

## 2. Experimental Details

A DJS-292 potentiostat with a standard three-electrode system was used for Cu_2_O octahedra deposition. An Ag/AgCl electrode in a saturated KCl solution was used as a reference electrode and a platinum coil as a counterelectrode. After rinsing several times with acetone and deionized water, Al foil was used as a working electrode.

The electrolyte solution consisted of 0.4 M cupric sulfate and 3 M lactic acid (LA). By complexing with the lactate ions, the copper ions were stabilized. The pH of the solution was adjusted to 9 by 4 M NaOH solution, and a dark blue solution was obtained under constant stirring. The electrochemical deposition was performed potentiostatically with a potential of −0.3 V (versus Ag/AgCl) for 30 min and the temperature of the electrolyte was kept at 60°C by water bath. In order to analyze the effect of other anions, CuSO_4_ was replaced with CuCl_2_ with the other parameters unchanged. The specimen for transmission electron microscopy observation was prepared by evaporating a drop of the dispersion onto a carbon-film-coated copper grid.

Morphology examinations were performed using XL30 S-FEG scanning electron microscope (SEM). Selected-area electron diffraction (SAED), bright-field (BF) imaging, and energy dispersive X-ray spectroscopy (EDS) analyses were carried out using a JEOL JEM 2100F transmission electron microscope (TEM) operating at 200 kV. Electron energy-loss spectroscopy (EELS) was carried out on a CM200 FEG transmission electron microscope operating at 200 kV.

## 3. Results and Discussion

**Figure 1:**
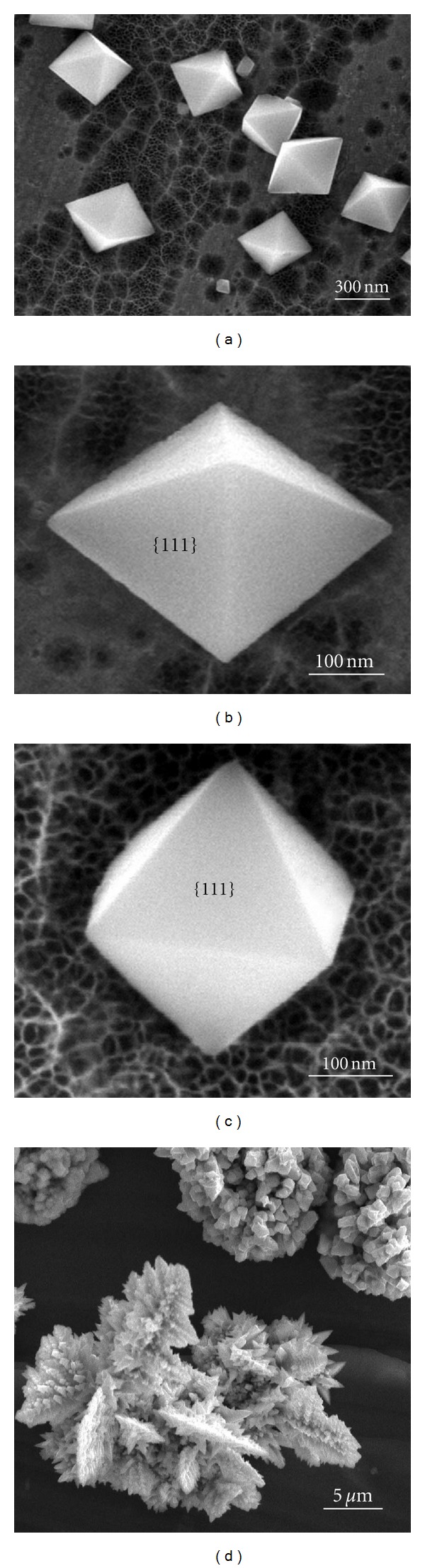
(a) Low-magnification SEM image of as-deposited specimen; (b) and (c) enlarged SEM images of as-deposited specimen; (d) SEM image of as-deposited specimen without lactic acid in the electrolyte.

The morphology of the as-deposited products was characterized using SEM. We recorded more than 150 nanoparticles and carried out a statistical analysis of the nanoparticles which showed that the average edge length of the octahedra is 300 ± 10 nm. It can be seen clearly from the low-magnification SEM image (Figure 1(a)) that the shape of the particles is a regular octahedron. The octahedra have slightly different orientations due to the anisotropy of the rough surface of Al foil. The enlarged SEM images of the octahedra viewed from different directions are shown in Figures 1(b) and 1(c), which depict clearly that the particles are perfect octahedra with eight {111} planes. The edge lengths are 300 and 295 nm, respectively. When increasing the deposition time to 1 h, no evident change of the octahedra size was observed. The octahedron looks like a pyramid (Figure 1(b)) viewed from the top and a rhombus (Figure 1(c)) from the side. Careful examination of the SEM images shows that there are lots of pores or network on the Al substrate surface after the deposition, which could result from the erosion of the base. In order to clarify the role of lactic acid in the formation of Cu_2_O octahedra, we also carried out the electrochemical deposition without addition of lactic acid in the electrolyte. When no lactic acid was added in the electrolyte, the flocculent precipitates were produced rapidly after the addition of NaOH solution into the CuSO_4_ electrolyte and the morphology of the as-deposited products changed drastically, which is shown in Figure 1(d). Two morphologies, one being aggregates composed of irregular particles, and the other being dendrites, can be found in Figure 1(d). This suggested that the lactic acid has a great influence on the formation of Cu_2_O octahedra. When CuCl_2_ was used as a copper salt in this experiment, the shape of most particles will become irregular. To determine the chemical composition of the octahedra, energy dispersive X-ray spectroscopy (EDS) was performed. The typical EDS spectrum from the particles in Figure 1(a) indicates that the particles are composed of Cu and O. The quantification of the spectrum shows that the ratio of Cu and O is close to 2 : 1, which suggests that the particles have a chemical formula of Cu_2_O.

**Figure 2:**
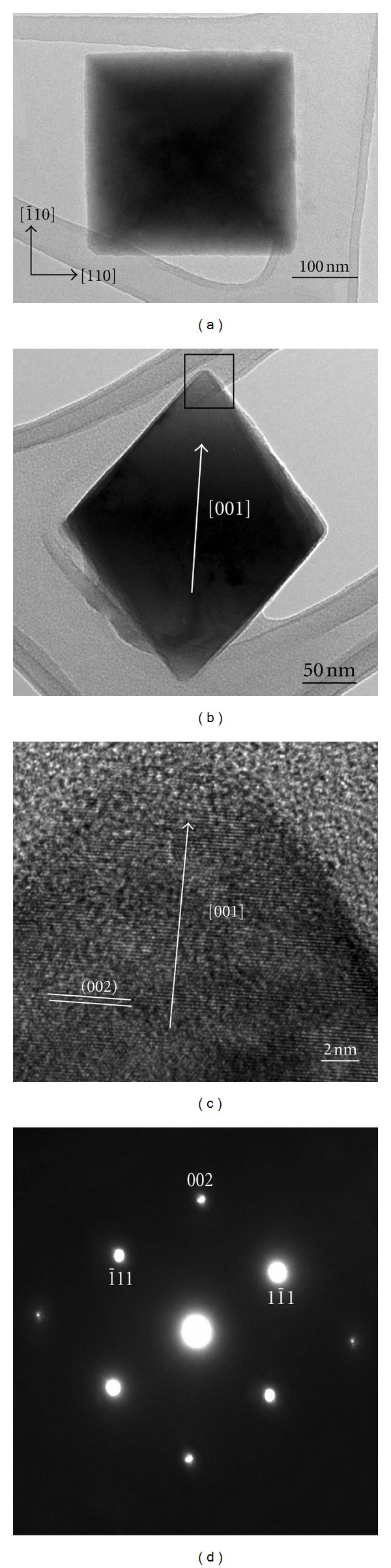
TEM images of individual Cu_2_O octahedron viewed from the top (a) and the side (b); (c) typical HRTEM image of the region enclosed by a square in (b); (d) corresponding SAED pattern.

The morphology and microstructure of the Cu_2_O octahedra were further characterized using TEM. Typical TEM images are shown in Figure 2. Most of the nanooctahedra look like a square (Figure 2(a)) and a rhombus (Figure 2(b)) in the projected images, which correspond to Figures 1(b) and 1(c). For the octahedra with eight {111} planes, the line connecting any two corners that are not in the same face is along the 〈100〉 direction. In Figure 2(a), we assume that the direction perpendicular to the paper outwardly is along [001] direction, then the two adjacent edges of the square that are perpendicular to each other are along $\left[\overset{-}{1}10\right]$ and [110] directions, respectively. In Figure 2(b), the rhombus is obtained viewed from the side of the octahedron. It can be seen clearly that two diagonal lengths of the rhombus are not equal, of which the longer diagonal is upward. HRTEM imaging was carried out to determine the growth direction of octahedra.  Figure 2(c) shows a typical HRTEM image of the region enclosed by a square in Figure 2(b). The interplanar spacing is measured to be 2.21 Å, which corresponds to the (002) crystal plane of Cu_2_O. The SAED pattern (Figure 2(d)) taken from an individual octahedron in Figure 2(b) suggests that it is single crystalline. Careful examinations of Figures 2(b)–2(d) show that the growth direction of Cu_2_O octahedra is along 〈001〉, which is indicated by a white arrow. This agrees well with the work of Golden et al. in which the preferred orientation of the Cu_2_O films is along 〈100〉 direction when deposited at pH = 9 [23]. The diffraction spots in Figure 2(d) can be indexed as $\left(1\overset{-}{1}1\right)$ , (111), and (002), respectively, using the lattice parameter of Cu_2_O (*a* = 4.2696 Å), which is in good agreement with the EDS analysis result. In a typical EELS spectrum acquired from a single octahedron, two peaks at 530 and 932 eV correspond to O-K and Cu-L_2,3_ edges, respectively. No impurity phase could be found in the products.

**Figure 3:**
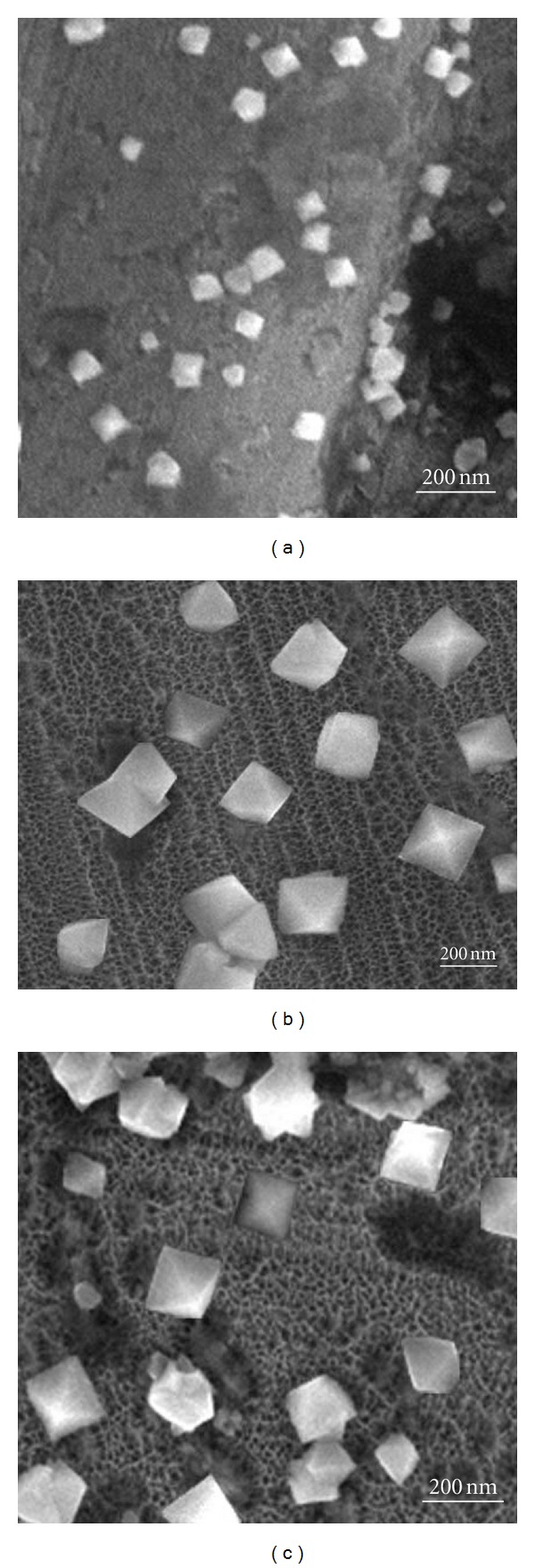
SEM images of the as-deposited specimen with different mole ratios of lactic acid/CuSO_4_: (a) *R* = 0.75/0.4; (b) *R* = 4.5/0.4; (c) *R* = 6/0.4 while other parameters are kept constant.

Further study shows that the shapes of particles are all octahedra in the presence of lactic acid, and SEM images are shown in Figure 3.  Figure 3(a) shows that the average size of the octahedra is 63.5 nm when the ratio of lactic acid and CusO_4_ is 0.75/0.4. As the ratio decreases to 1.5/0.4, the size rises to 105 nm; but when the ratio increases up to 4.5/0.4 and 6/0.4, the size decreases to 167.5 and 132.5 nm (Figures 3(b) and 3(c)), respectively. This suggests that the size of the octahedra increased initially and then decreased with the increase of the concentration of the lactic acid. 

**Figure 4:**
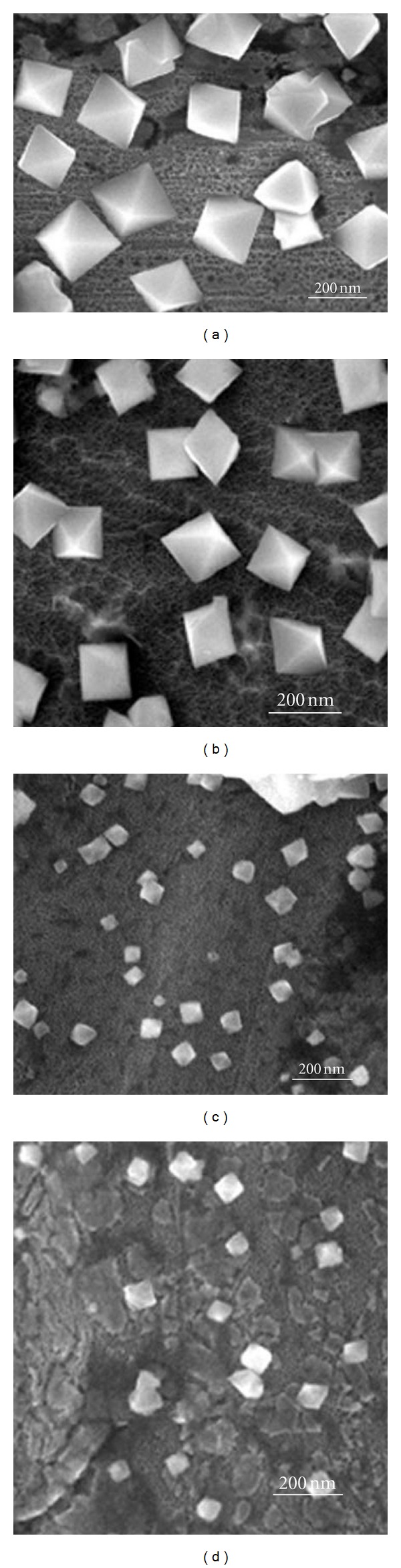
SEM images of the as-deposited specimen at different bath pH: (a) pH = 8; (b) pH = 9; (c) pH = 11; (d) pH = 12 at −0.3 V for 30 min.

In order to clarify the effect of pH, a bath with different pH was used to fabricate the Cu_2_O nanoparticles.  Figure 4 shows the SEM images of the octahedra deposited at different pH. It suggests that the octahedra can form in alkaline solution if the other reaction parameters are kept constant. At pH = 8, the average size of the octahedra in Figure 4(a) is about 382 nm, which is larger than others. When pH rises to 9, the size is approximately 300 nm (Figure 4(b)), relatively smaller than that deposited at pH = 8. However, when the pH values are up to 11 and 12, the size of the octahedra has an obvious decrease compared with those obtained at lower pH, which were about 92 and 87 nm, respectively. From the typical EDS spectrum, it is found that the particles have a chemical formula of Cu_2_O.

**Figure 5:**
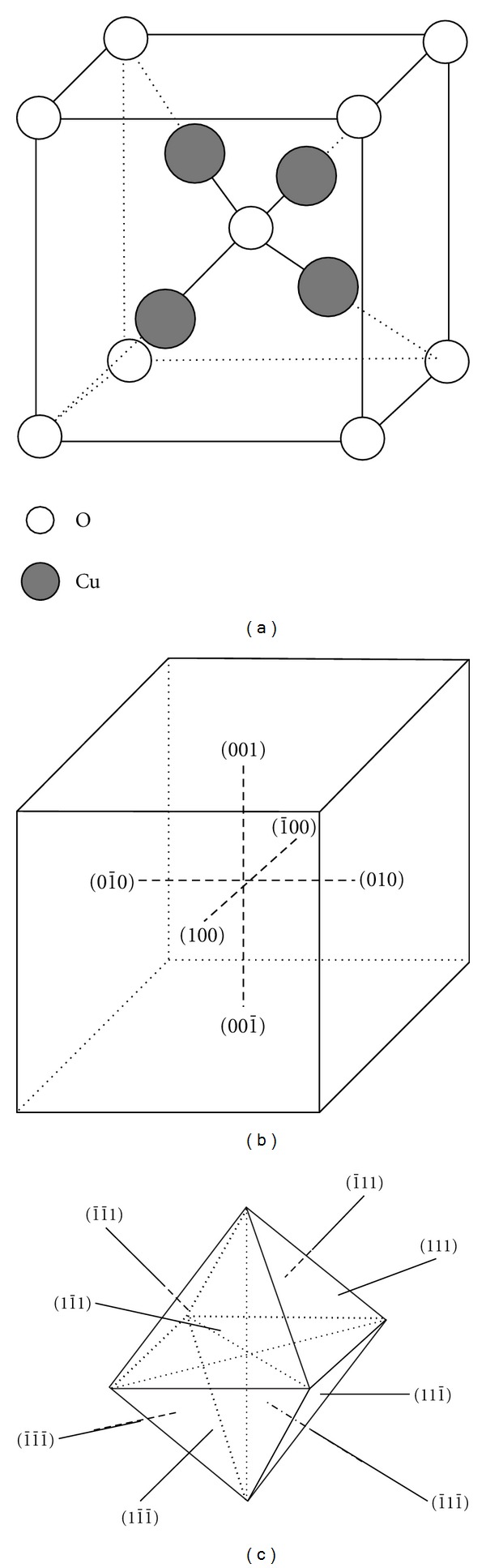
(a) Crystal structure of cubic-phase Cu_2_O; (b) and (c) schematic illustration of the crystal faces of Cu_2_O cube and octahedron.

In the crystal structure of Cu_2_O shown in Figure 5(a), the oxygen atoms are arranged in a body-centered-cubic manner, with each oxygen atom being surrounded by a tetrahedron of copper ions, each of which has two oxygen neighbors. The {100} and {111} surfaces in cubic Cu_2_O are different in the surface atom structures and bonding as well as the possibility of chemical reactions. It is believed that during the crystal growth process, the crystal faces with higher growth rate will be eliminated first and the morphology is defined by the crystal faces with the slowest growth speed. The shape of the crystals was determined by the ratio (*R*) of the growth rate along the 〈100〉 direction to that along the 〈111〉 direction [24]. The preferential crystal growth along the 〈111〉 direction leads to the formation of the nanocubes [14], while the high growth rate of {100} planes leads to the formation of the octahedra [25]. This is illustrated in Figures 5(b) and 5(c). 

Lee et al. [26] deposited spherical Cu_2_O clusters onto Al substrate in acidic solutions, and Cu_2_O thin films were also synthesized on Al foils. Leopold et al. [27] discussed the reaction scheme of Cu_2_O nanocrystals deposited on coppers for the self-oscillations and explained the sudden decrease in the potential and increase in the local pH with precipitation model. 

Based on the previous work and experimental results, we proposed a growth mechanism for the Cu_2_O octahedra. The electrode reactions can be described as follows [27]:

(1)CuL22−+OH−→[CuL2(OH)]3−(2)2CuL22−+2e−+2OH−→Cu2O(s)+4L2−+H2O(3)2[2CuL2(OH)]3−+2e−→Cu2O(s)+4L2−+H2O(4)L2−+H2O→HL−+OH−

**Figure 6:**
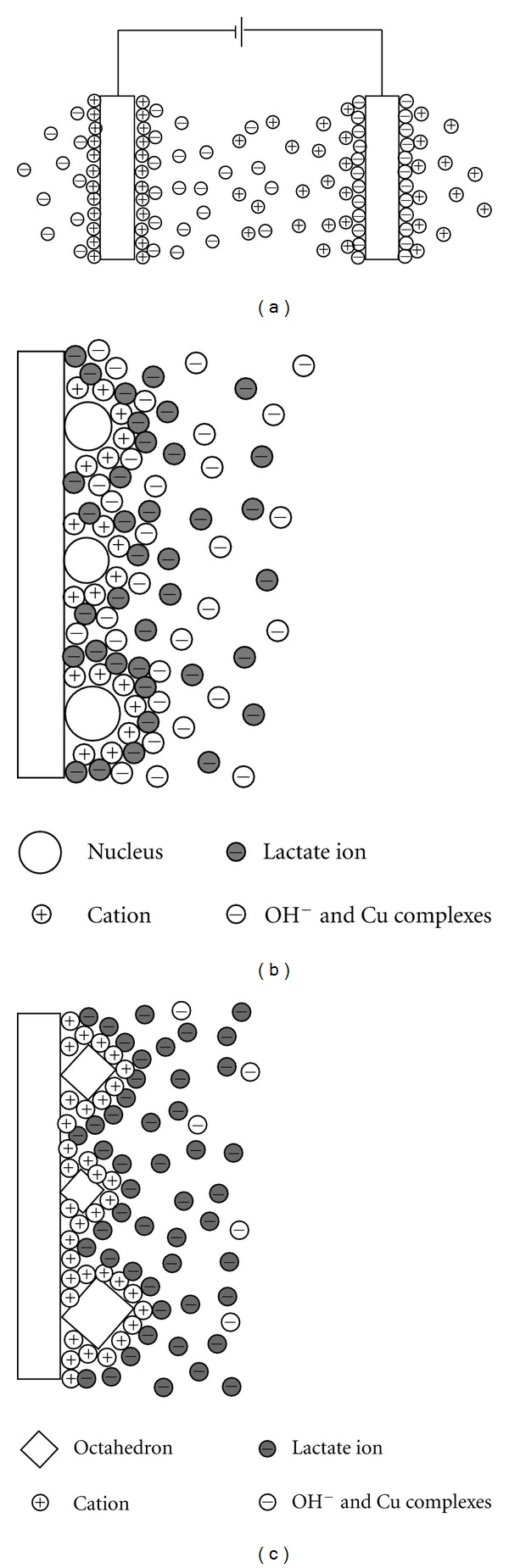
(a) Schematic diagram of the adsorption process on the electrodes; (b) and (c) formation process of Cu_2_O nuclei and octahedra.

The formation process of Cu_2_O octahedra is shown in Figure 6. At the initial stage, under the effect of the electric field and Coulomb force, the double layer can form on the electrodes (Figure 6(a)). In alkaline solution, the Cu(II) has two forms in the presence of lactic acid, one being CuL_2_^2−^ and the other being [CuL_2_(OH)]^3−^. The Cu(II) complexes in the diffusion layer were reduced into Cu_2_O (reactions (2) and (3)) and the L^2−^ ions were liberated. Then, the Cu_2_O adsorbed on the surface of the cathode and nuclei formed in an extremely short time (Figure 6(b)). The lactate ions, as a face selective adsorption additive [28], could be adsorbed on the {111} planes and confines the crystal growth along 〈111〉 directions. Compared with {111} planes, the {100} planes grew faster. At the same time, the lactate ions could be protonated and OH^−^ ions were released (reaction (4)), which could balance the local pH close to the cathode to a certain extent. In this process, SO_4_^2−^ [17] and OH^−^ ions might play important roles as the face selective adsorption additives to control the final shape of the Cu_2_O crystals. Therefore, the {100} planes were eliminated and finally Cu_2_O octahedra were formed. Then the ripening mechanism was dominant in the subsequent steps, which led to the size increase of Cu_2_O octahedra. With the increase of the time, the diffusion layer became thicker and the concentration of lactate ions in the diffusion layer increased. The size of the octahedra was gradually stabilized because no enough Cu(II) complexes in the diffusion layer could be reduced and no extra Cu_2_O compounds were formed on the surface of the octahedra, which is demonstrated in Figure 6(c). As the depletion of OH^−^ ions, the pH value of the electrolyte tended to be neutral (pH = 7.5 after the deposition for 30 min) and the reaction will be terminated.

## 4. Conclusions

Single-crystalline Cu_2_O octahedra have been deposited onto the Al foils by a simple electrochemical deposition method. The Cu_2_O octahedra have an average edge length of 300 ± 10 nm. The growth direction of Cu_2_O octahedra is determined to be along the [001] direction. The size of the octahedra firstly increases and then decreases with the increase of mole ratio of lactic acid/CuSO_4_, while the size of the octahedra decreases with pH. The Cu_2_O octahedra can be obtained through the synergic effect of face selective additive adsorption upon the particle growth and ripening mechanism with the change of the pH.
